# Risk Assessment of the SME Sector Operations during the COVID-19 Pandemic

**DOI:** 10.3390/ijerph18084183

**Published:** 2021-04-15

**Authors:** Katarzyna Grondys, Oliwia Ślusarczyk, Hafezali Iqbal Hussain, Armenia Androniceanu

**Affiliations:** 1 The Management Faculty, Czestochowa University of Technology, 42-201 Czestochowa, Poland; katarzyna.grondys@pcz.pl (K.G.); olislusarczyk@gmail.com (O.Ś.); 2 Geneva School of Economics and Management, University of Geneva, 1205 Geneva, Switzerland; 3 Taylor’s Business School, Taylor’s University, 1 Jalan Taylors, Subang Jaya 47500, Malaysia; 4 University of Economics and Human Sciences, 01-030 Warszawa, Poland; 5 Faculty of Administration and Public Management, The Bucharest University of Economic Studies, 010374 Bucharest, Romania; armenia.androniceanu@man.ase.ro; 6 Research Department, University of Social Sciences, 90-113 Lodz, Poland

**Keywords:** COVID-19, risk management, SME risk, risk factors

## Abstract

The subject matter of the article relates to the assessment of the perception of selected types of risk in economic activities of the SME sector, which change their intensity as a result of the outbreak of the COVID-19 pandemic. The current economic downturn is unprecedented and involves many companies and industries that have faced new, previously unknown challenges and threats. The objective of the article is to identify the most important risks and their resources based on the empirical research carried out in small and medium-sized enterprises in Poland. The formulated objective was accomplished using the data collection method, i.e., the survey and reports on the condition of the SME sector in Poland as well as statistical data analysis methods, i.e., structure index and the analysis of variance, using the SPSS system. The process of primary data collection was carried out by means of an electronic survey among selected enterprises of the SME sector, conducting business activities in Poland. In the study, the employment factor was taken into account as a determinant of the perception and assessment of the intensity of selected risks arising from the economic activity in the Polish market in the conditions of the current economic downturn. On the basis of the obtained results, the impact of market, economic, financial and operational risks, depending on their intensity, on the functioning of micro-, small and medium-sized enterprises was identified. Based on the analysis of variance, the effect of the size of the company on the level of individual risks was also examined. As a result of the observations made, it was established that, during the pandemic, the level and type of risk is similar in all the surveyed enterprises. They are most often threatened by strong competition in the industry, an increase in energy prices and insufficient profit. The overall results of the empirical research indicate the importance and the need to manage the key threats to the Polish SME sector.

## 1. Introduction

The risk of the pandemic is nowadays the key challenge the whole world is facing. Its major consequence, among others, is the global economic crisis which threatens many, particularly smaller, enterprises. The COVID-19 pandemic has significantly affected the reduction in the income and employment level of many companies. The suspension of economic processes carries serious consequences of the temporary or permanent disappearance of business [1,2]. Management boards of nearly all organizations have confronted the need to make a series of difficult decisions while facing a sudden crisis caused by the pandemic. In such a situation, an intuitive assessment of the results of risks most frequently accompanying smaller enterprises is definitely not enough. Dealing with a number of unpredictable events is a general risk of conducting an economic activity. According to the previous research [3,4], risk management helps to improve the efficiency and competitiveness of enterprises in the dynamically changing environment.

At the same time, the sector of small and medium-sized enterprises is responsible for the development of most national economies. In the European Union alone, 98% of all enterprises belong to the SME sector, providing 67% of the total employment and generating 58% of gross value added [5]. In Poland, this sector is responsible for almost half of the generated GDP.

Small and medium-sized enterprises are the most vulnerable group of entities since they do not have the resources to survive the crisis [6,7]. In terms of industries, the companies with the most serious problems with the continuity of supply and decline in demand are the ones in the area of passenger transport, tourism or services [8]. On the other hand, existing small and medium-sized enterprises are seen as crucial for the economic development of each country [9,10,11,12] and they symbolize the natural signal of social entrepreneurship, which large operators need to unleash within themselves [13,14]. The functioning of small and medium-sized entities brings a number of benefits, beginning with the decentralization of the economy and equal development of regions by increasing the level of innovation of the country and ending with mitigating crises. At the same time, the highly variable economic environment intensifies uncertainty and unpredictability of economic phenomena and thus increases the risk related to conducting a business activity [15]. Moreover, the size of the company affects the level of the risk taken, which is lower in larger enterprises. Small-sized companies are exposed to a higher risk three times more than large businesses. In turn, the risk for medium-sized companies is lower than for small ones, but higher than for large enterprises [16].

One of the current crises is the reduction and exclusion of economic activities due to the COVID-19 pandemic, which clearly affects the development of small and medium-sized enterprises by inhibiting their expansion, worsening their financial situation and ultimately contributing to their insolvency and bankruptcy [17,18]. Hence, risk management treated as a mechanism to prevent or reduce financial losses and improve the services provided [19], takes on a new meaning in the light of the impact of unforeseen factors.

Pre-pandemic data indicate that the survival rate of the SME sector companies is one of the lowest among all enterprises [20]. This is confirmed by Xu B. et al. [21], in the opinion of whom the SME failure rate is very high, even though they represent a significant part of the market and generate a high level of GDP. According to the latest report by PARP (The Polish Agency for Enterprise Development) on the condition of small and medium-sized enterprises in Poland, both the level of newly established companies and the number of liquidated entities indicate slight upward trends [22,23]. In connection with the outburst and restrictions caused by COVID-19, not only Polish enterprises are facing unprecedented negative effects of their operations [24,25,26].

Micro-, small and medium-sized enterprises are exposed to numerous external and internal threats more than large ones. In the assessment of the functioning of small and medium entities it is important, therefore, to recognize the conditions and factors of survival, and also to look at the limitations to their activities from the point of view of systemic risk management [27], in which the risk of the pandemic is inscribed. The objective of the article is to assess business risk in micro-, small and medium-sized enterprises arising due to the COVID-19 pandemic. Moreover, the aim of the conducted research is to help decision-makers and practitioners to develop strategies to respond to the effects of the pandemic seen in the SME sector.

## 2. Literature Review

### 2.1. Key Risks of Economic Activity

Risk is a fixed component of various areas of the enterprise and concerns the uncertainty of events and their future effects on the condition of the entity, which may relate to both internal activities and the environmental impact. Risk is defined as the measurable probability of loss or reduced expected return [28]. Effective risk management requires the identification and assessment of a possible risk in the specific economic entity, thus research was conducted to analyze the health security data from the period prior to the detection of COVID-19 in December 2019 [29]. Effective risk management requires the identification and assessment of possible risk occurring in the economic entity. The SME sector, compared to larger enterprises, uses less economies of scale and has less access to resources [30]. Moreover, due to the low level of the equity ratio, it is more exposed to external influences [31]. This indicates that the survival of the SME sector entities is a serious challenge, which requires development as well as the application of risk management models. This allows the identification of key risks and handling them effectively before some serious consequences occur [32].

Ch. Chang et al. [33] using arithmetic, geometric and harmonic means, assessed how individual countries could prepare for COVID-19 and how, in relation to the above, effective business, economic and financial decisions can be planned. In turn, G. Wang et al. [34], when considering risk management, focused on economic and social development provided by universities which, in crisis situations, gather relevant human resources necessary for emergency medical services. This is illustrated by one of the behavior models in the crisis situation related to the pandemic, which is different in the case of enterprises where human resources are reduced. In turn, C.M. Hall et al. [35] presented the impact of the COVID-19 pandemic on the tourism industry, and more specifically the limitations to its development. The research results have led the authors to the identification of the direction of global changes in the context of sustainable development. Business risk itself is defined in the literature as the aggregation of events and phenomena or residual risk [36]. It is also perceived as a nonsystematic risk in the context of the valuation of assets. Business risks vary in nature and arise due to a number of factors of internal and external origin [37]. Standard Bank (one of the largest banks in South Africa) has published 10 major threats to small and medium-sized enterprises, including public opinion, cash flows, supply chain, business interruption, loss of key partners, regulations and provisions, intellectual property, data security, business assets, and human capital [38]. In some enterprises, risk management is only a part of extraordinary events, in others, risk management is applied too late, dealing with consequences rather than prevention. These threats are inscribed in the commonly identified and analyzed business risk, the categories of which primarily include operational risk, market risk, economic risk, and financial risk [37] and which are the subject of the empirical research in this article.

### 2.2. Operational Risk in Activity of SME

Operational risk generates potential losses resulting from the entity’s inadequate or defective logistic system, including processes and resources [39]. W. Hemrit and M. B. Arab [40] define operational risk as full, which arises within the organization and is influenced by all its elements but is also the result of external factors. According to the research carried out by L. Allen and T. Bali [41], operational risk is less common than other risks, but it may have more serious effects for the entity’s operations. According to the Insurance and Reinsurance Act, market risk means the possibility of incurring losses due to fluctuations in the level and volatility of the market prices of assets, liabilities and financial instruments [42]. In the case of economic activities, this risk includes business relationships with suppliers and customers, which, on the one hand, are the basis for the entity’s operations and, on the other, may pose a threat to it. Since many SMEs and microenterprises do not record sufficient cash flows in relation to the assumed plans, they bear financial risk [43]. According to the guidelines by Basel and Solvency Risk Assessment Standards in Banks and Insurance Companies, financial risk is unique in its nature and can be defined as any event or operation which may adversely affect the ability of the organization to accomplish objectives or implement the strategy [44]. This means that the funds collected in these entities are mainly used for the implementation of current operations, therefore any increase in costs results in a reduction in the budget. In such a situation, when the budget is not well planned and cash generation does not match the forecast, the company faces financial problems. Economic risk is associated with the risk of conducting a business activity in the country. Its important component is tax risk which includes threats related to variability of tax law, variability in interpretation of existing legislation and methods of tax construction. It is also important to mention the level of interest rates or changes in labor rights, which significantly affect the profitability of the entity [45]. Moreover, financial risk is a frequent obstacle and limits the development of SMEs [29]. J.C. Alves et al. [46] claim that small-sized enterprises are more vulnerable to the risk associated with the current crisis and at the same time have limited insight into the effective handling of its long-term effects. The poor condition and risk of bankruptcy of small-sized enterprises are due to a sudden drop in market demand. On the other hand, these companies show greater flexibility in responding to economic problems. An important element in the prediction of individual business risks is to constantly monitor the impact of the pandemic on business operations [47,48] and to undertake investments for both structural and nonstructural measures [49].

Risk assessment is an important stage of the risk management process. At the same time, the pandemic situation has caused the complexity of operational, financial and market processes in the face of the operating conditions to be even larger, and the arising threats, which intertwine, complicate the process of handling them (Bazylea II) [50]. This contributes to an increase in the significance of the risk management process and constitutes a great challenge to the function of risk management. The need to manage risk is also evident in EU documents. Pursuant to Article 86 of Directive 2013/36/UE, an organization should have a specific strategy and related procedures for the identification, measurement, management, and control of financial risks to ensure its financial security [51].

### 2.3. Small Business Activities in a Pandemic

The COVID-19 pandemic has contributed to an increase in the probability of individual business risks [52,53]. Their minimization in this respect requires a new approach to systemic risk management which includes pandemic risk. The time and manner of responding to the spread of the virus in individual countries of the European Union has caused complex and widespread negative economic consequences which are shaping the current direction of development of social and economic processes [54,55].

Industrial companies (59.9%) and hotels and restaurants (59.6%) are particularly threatened by economic slowdown. A relatively large proportion of these companies bear the risk of losing their financial liquidity and even bankruptcy respectively 39.4% and 38.5%). For 45% of enterprises, weakening demand becomes the problem not only in terms of development but mostly in terms of a financial and economic situation. The consequence of economic slowdown is growth in competition in the market, which is the greatest obstacle to the development and sustainability of the economic and financial situation of SMEs. Additionally, macroeconomic conditions, i.e., inflation, interest rates, exchange rate constitute a significant threat to the activities of the surveyed companies. Almost 50% of SMEs indicate they are not able to establish appropriate relationships with customers, which affects the risk of failure to meet customers’ expectations [56].

The outbreak of the pandemic in 2020 affected the change in the perception of business risk and its management [57,58]. In most countries, in order to sustain the activity of enterprises, various types of financial tools are used, the task of which is to finance a part of, or all the costs incurred due to suspension, closure or slowdown in the operations of enterprises [59]. The primary objective of this type of support is to sustain the economic activity of small-sized enterprises through financial coverage of potentially lost profits as a result of minimizing the risk of economic depression [60]. Many countries have developed rapid solutions to support the operations of the SME sector, i.e., direct financing, tax reliefs, financial guarantees, low interest loans, etc. As a part of minimizing individual risks associated with the activities of enterprises, the following solutions of government support for the national economy have been successively implemented [61,62,63]:

1. Financing a part of the expenditure related to job protection and protection against the loss of income of various professional groups; securing financial security of companies and introducing bank guarantees ensuring their solvency—PLN 212 billion;
2. Providing additional support to companies regardless of their size and industry in order to maintain employment and protection—PLN 100 billion;
3. Exemption from social security contributions for micro- and small entrepreneurs and idle time pay for sole proprietorship.

During the pandemic the differences are even more obvious. According to the results of the latest report—SME Scanner [64]—at the end of 2020 43% of micro-, small and medium-sized enterprises did not recover their level of turnover from the beginning of that period. Many of these companies will not survive another year in the market due to the coronavirus. This is confirmed by the research conducted by the Confederation Lewiatan, the objective of which was to assess the threat to the economy due to the COVID-19 pandemic. The obtained results indicated that 94% of the surveyed companies experienced the impact of the pandemic and restrictions implemented as a result of it, and more than half of these companies view the changes taking place as very serious. At the same time, the smaller the company the greater the negative consequences of the pandemic impact on the situation of the entity [60]. According to the “COVID-19 Business Pulse Survey (COV-BPS)–Poland” report, 52% of enterprises recorded a decrease in sales within 30 days prior to the survey, compared to the same period in 2019. Over 70% of companies are coping with a decline in revenues at higher or unchanged costs or with unchanged revenues at higher costs [65].

This translates into the ability to handle business risk which is developed less in smaller entities: large companies are more likely to plan a reduction in employment whereas smaller ones are considering the closure of their operations. Moreover, along with the prolonged pandemic, the number of companies facing financial difficulties is increasing. The available government support is insufficient; therefore, the SME sector is making an attempt to reduce the risk of bankruptcy due to the COVID-19 pandemic independently. Among the measures taken to stay in the market, the following can be found: securing material stocks, providing new services, searching for new markets, or increasing Internet sales [66].

## 3. Materials and Methods

In order to examine the level of market, economic, financial, and operational risks due to the pandemic and the related restrictions, the survey was carried out among micro-, small and medium-sized enterprises conducting their business activities in Poland in the period of the second quarter 2020. The SME sector in Poland generates almost half of GDP (49.1%). It includes the vast majority of enterprises in Poland—99.8%. Among them, the largest group (96.7%; 2.08 million) is microenterprises [67].

Based on the REGON number of these enterprises, the size of the research sample was determined at the level of 496 entities for α = 95% and a maximum error of 5%. The survey questionnaire was sent electronically to randomly selected enterprises, among which there were micro- (60.3%), small (28.6%) and medium-sized enterprises (11.1%). Due to the scope of the questions, the obtained responses came from employees in managerial positions or business owners. Four main explanatory variables relating to the sources of individual business risks, i.e., market (X_1_), financial (X_2_), economic (X_3_), and operational (X_4_) were identified in the research. The task of the surveyed enterprises was to assess the intensity of the impact of certain phenomena and the related individual risks on the activities of their companies. The questions included in the questionnaire were closed-ended ones and the assessment of the studied phenomena was expressed on the Likert scale from 1 to 5, where 1 was the minimum intensity and 5 was the maximum intensity of the risk impact. Selected descriptive statistics for each variable were analyzed in the research—absolute frequencies, mean and bar charts. The results were presented using the structure index and the arithmetic mean. Additionally, the relationships between the intensity of risk and the company’s experience were examined in this field using the nonparametric Spearman rank correlation test and Pearson’s Chi-square.

In order to determine the impact of the size of the company on the level of intensity of individual risks, the analysis of variance was conducted using the analysis of variance. The ANOVA analysis was to test the significance of differences between means for the surveyed groups of enterprises due to the assessment of individual risks [68]. The analysis was also used to assess the risk of investment management [69]. The conditions for conducting the analysis were checked based on the homoscedasticity test for the key risks, Levene’s test and normality test, where the significance level-p must be equal or greater than the value of 0.05. In order to compare the mean value of the specific variable for independent tests, the nonparametric Kolmogorov–Smirnov test was used [70].

## 4. Results

### 4.1. Risk in Activities of Micro-, Small and Medium Enterprises in Poland

The level and type of risk in Polish enterprises are conditioned by various external and internal factors. In this respect, several studies have been carried out on barriers and obstacles that increase the risk to the development of small and medium-sized enterprises in Poland [71,72,73]. The main problem for the surveyed entities is very high taxes and other financial burdens and their complexity (over 60% of indications). Another problem is too excessive costs related to conducting operational activities in terms of the costs of employment or purchase of materials and raw materials (44%). The surveyed sector also often copes with untimely payment of receivables by customers and difficulties in obtaining external funds (42%). In turn, among general economic barriers, entrepreneurs most often indicate the uncertainty of the economic and political situation in the country, which increases the risk of operational activities, in particular in terms of the development of innovation and investments (48%). On average, a third of Polish enterprises also have problems with a shortage of long-term financing and working capital. In the face of the COVID-19 pandemic, nearly 90% of companies in Poland have recorded disruptions in their activities due to social isolation and changes in customer behavior, which resulted in a decline in demand and supply chain disruptions [74].

**Figure 1:**
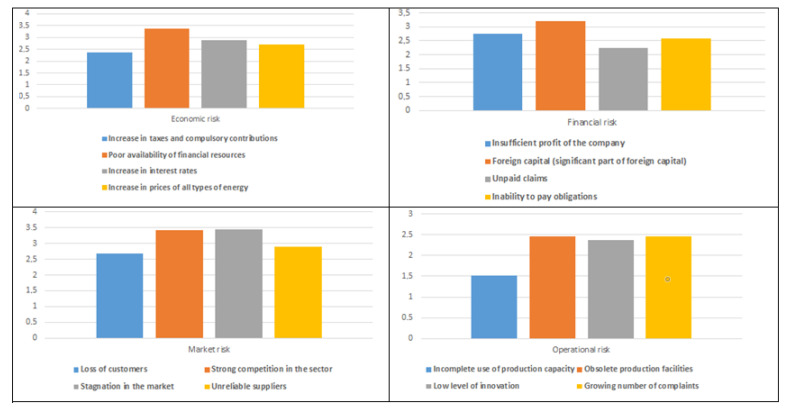
Average rating of the intensity of market, economic, financial, and operational risks in the surveyed enterprises as a result of the pandemic risk.

The overall average level of risk assessment, regardless of the size of the company in the surveyed sample is collectively presented in Figure 1.

The data in the individual diagrams indicate that during the pandemic none of the analyzed risk groups is on average higher than 3.5, i.e., it is at an average level. The highest average ratings include the factors of market risk, where the minimum amounts to 2.5. In turn, the lowest average ratings relate to operational risk, where the minimum value amounts to 1.5. Small and medium-sized enterprises most frequently fear an increase in the level of market risk due to the pandemic, which results in an increase in stagnation in the market and a reduction in the level of sales. In turn, they consider the factors related to the efficiency of their activities to be less risky since they are fully dependent on the strategies taken and the manner of management of the entity. On the other hand, taking into account only individual risk factors, the largest impact on the activities of enterprises is exerted by stagnation in the market (type of market risk), and the least impact—by incomplete use of production capacity (operational risk).

### 4.2. Level of Risk Intensity Depending on the Size of the Entity

#### 4.2.1. Market Risk

In the subsequent part of the paper, the assessment and impact of individual risks on the business activity is examined, broken down by the number of employees in the entities. In Table 1, Table 2, Table 3 and Table 4, the level of risk intensity is presented for each group of the surveyed companies broken down by the number employees, where:

- low intensity was the percentage of ratings of 1 and 2 (very low and low risk),
- high intensity was the percentage of ratings of 4 and 5 (high and very high risk).

In the case of half of the surveyed companies broken down by their size, the sources of market risk with the highest intensity are primarily the loss of customers and strong competition in the sector. During the pandemic and the threat of the greatest restrictions, the ability of companies to survive is diminishing. Moreover, the surveyed enterprises are definitely the least likely to fear business risk due to unreliable suppliers. In turn, stagnation in the market constitutes a high level of threat for nearly half of medium-sized enterprises, whereas this level is rather low for other entities. When analyzing the highest percentage of ratings due to the level of risk intensity, the following conclusions ought to be drawn:

1. Microenterprises are the least afraid of stagnation and unreliable suppliers;
2. Small-sized entities most often bear the risk of strong competition, whereas they are the least afraid of stagnation in the market;
3. Medium-sized enterprises most often bear a high market risk related to the loss of customers, competitive environment and stagnation in the industry, however, they are far less concerned about unreliable suppliers.

The obtained results prove that market risk determined by the environment of customers, suppliers and competitors is perceived as strongly affecting the activities of the surveyed enterprises in the period of imposed restrictions, related to the spread of COVID-19. This requires directing all the company’s forces to fight for its customers against the competitive environment. At the same time, this makes the area of customer service to be crucial for ensuring the continuity of the entity’s operations. Consequently, this forces the necessity to take actions aimed at acquiring and retaining customers as well as continuously improving the core process based on innovative solutions to counteract competition. During the pandemic, minimizing market risk by enterprises primarily consists in expanding their commercial offer and searching for new ways to reach existing and new customers.

#### 4.2.2. Economic Risk

The highest risk due to the pandemic is experienced by the surveyed enterprises in the area of an increase in prices of all types of energy. On the other hand, the surveyed entities are far less afraid of the risk of availability of financial resources, an increase in interest rates and taxes and contributions.

A significant part of micro- and medium-sized entities are not concerned about the risk related to an increase in taxes and compulsory contributions. In turn, regardless of the size of the operator, their activities are least threatened by poor availability of financial resources (grants, loans) as well as an increase in interest rates. The risk of an increase of individual costs has risen significantly during the pandemic. In 2021, one may observe increases in prices of energy and some taxes. On the other hand, the risk of access to financial resources and interest rates on investment loans have declined so that enterprises can survive a difficult period of downtime. When analyzing the highest percentage of ratings due to the level of risk intensity, the following conclusions ought to be drawn:

1. The surveyed enterprises are most often concerned about an increase in prices of all types of energy;
2. Both small and medium-sized entities are least afraid of an increase in taxes and compulsory contributions as well as an increase in interest rates related to the investments taken.

The obtained results indicate that economic risk has a particularly large impact on the functioning of enterprises in relation to an increase in the amount of taxes and compulsory contributions. Increasing energy prices are also not less indifferent. At the same time, it should be noted that these factors are independent of the internal activities of the entity, therefore, any actions taken to minimize these phenomena may be ineffective. Shaping these factors is determined by the political and economic environment of the country, thus entities, while conducting their activities must consider the risk of an increase in taxes and energy prices in their long-term management strategy.

#### 4.2.3. Financial Risk

The data concerning financial risk (Table 3) show that, simultaneously, in the case of micro-, small and medium-sized enterprises, the risk of an increase in foreign capital (significant part of foreign capital), unpaid claims and inability to pay obligations (insolvency) is at a high level after the outbreak of the pandemic.

The risk factor of insufficient profit of the company is the most threatening financial risk factor for most of the surveyed enterprises, regardless of their size. At the same time, the surveyed companies also highly rate the risk of unpaid claims and inability to pay obligations (insolvency) during the pandemic. When analyzing the highest percentage of ratings due to the level of risk intensity, the following conclusions should be drawn:

1. Medium-sized enterprises are slightly more often than the others most concerned about generating insufficient profit during this difficult time;
2. Microentities more often than the others highly rate the occurrence of the risk of unpaid financial liabilities and insolvency.

The obtained results indicate that financial risk seemingly determined by financial results of the entity is largely shaped by the present economic situation. The objective to generate profit can be found as the key one in ensuring the continuity of the entity’s operations. During the pandemic, both micro-, small and medium-sized enterprises ought to primarily concentrate their efforts on increasing revenue and reducing operating costs.

#### 4.2.4. Operational Risk

Operational risk factors, i.e., incomplete use of production capacity, obsolete production facilities, low level of innovation, and growing number of complaints totally depend on actions taken within the company. The analysis of operational risk (Table 4) indicated many similarities for small and medium-sized enterprises. These entities, due to the spread of COVID-19, are most afraid of incomplete use of production capacity. In turn, for microentities, it is usually a low level of threat in their functioning. Other situations posing a threat to operating activities are significantly more often likely to affect the activities of all enterprises regardless of their size.

The manufacturing process implemented in the smallest entities is characterized by high labor-intensity and low capital expenditure, therefore, the machinery park of such enterprises is usually universal and flexible in changes in the direction of production [75]. Therefore, enterprises of this sector retain high flexibility in responding to the changing demand of customers and more often respond to individual customer needs [76]. Thus, the percentage of low ratings in the case of microenterprises is the highest. At the same time, it is small and medium-sized enterprises that experience the slowdown in demand more, which translates into their excess production capacity, which is not used during the pandemic.

### 4.3. The Analysis of the Impact of the Size of the Company on the Level of Intensity of the Examined Risks

In order to determine the impact of the company’s size on the level of intensity of individual risks, the analysis of variance was carried out using the nonparametric Kołmogorow–Smirnow test. This allowed for verifying how the average of key risk assessment is shaped based on the number of employees in the enterprise.

On the basis of the results of the Kołmogorow–Smirnow test (where *p* > 0.05 for individual groups of the risks examined), it was identified that the distribution of the variable of market, financial, economic, and operational risks is different from the assumed theoretical distribution, which conditions the further analysis of variance—ANOVA (Table 5).

The results of the test F = 14.21 and *p* = 0.0063 are the evidence of the statistically significant result. It can be concluded that there is the relationship between the intensity of market risks and the size of the company. In the case of the analysis of financial risk, the value of the test F = 33.91 and *p* = 0.0571 should be found statistically insignificant, therefore, it can be concluded that there is no relationship between the intensity of financial risks and the size of the company. The analysis of variance of the intensity of economic risk using the parametric test F = 73.01 and *p* = 0.2383, therefore, it can be concluded that the examined relationship is statistically insignificant. It can be noted that there is no relationship between the intensity of economic risk and the size of the company. The results of the test F = 117.02 and *p* = 0.0310, thus, it can be concluded that the relationship in question is statistically significant. On the basis of the above, it can be noted that there is a relationship between the intensity of operational risk and the size of the company.

The results indicate that market and operational risks result from the number of employees in the company. Larger enterprises are more likely to assess market events and the ones related to basic processes as a threat to their activities, therefore, these entities ought to take more advanced manners and strategies of dealing with threats.

### 4.4. The Impact of the Experience of Enterprises in Risk Management on the Level of Individual Risks

In order to identify significant relationships between the company’s experience in risk management and the assessment of intensity of individual risks, statistical testing using nonparametric tests was carried out, i.e., Spearman rank correlation coefficient and Pearson’s Chi-square test. The experience of risk management included the examination of the following explanatory variables Y, where:

- Y_1_ means how long the company has been managing risk, where y_11_—yes, for less than a year; y_12_—yes, from 1 to 4 years; y_13_—yes, from 5 to 9 years; y_14_—yes, for more than 10 years; y_15_—the company does not manage risk;
- Y_2_ means that the position/person in the company is responsible for risk management, where y_21_—risk specialist; y_22_—company’s owner; y_23_—managers authorized by management; y_24_—department head; y_25_—no one manages risk.

In order to assess the relationships between the intensity of risk and experience in risk management the Spearman’s rho test was used since both variables are expressed on the ordinal scale. The summary results are presented in Table 6.

In each of the examined four areas of individual risks, at least one was identified, the intensity of which depends on how long the company has been managing risk and thus what experience it has in this field. In the case of market risk, one may observe a significant relationship between the risk of the loss of customers and risk management time for *p* < 0.0001 and rho = 0.45. This means that the wider the experience in risk management the more often the enterprises fear a shrinkage of their market. In the area of economic risk, one may observe a significant relationship between the rising prices of all types of energy and risk management time for *p* < 0.01 and rho = 0.32. This means that the wider the experience in risk management the more often the enterprises realize the rising prices of energy. In the area of financial risk, a significant relationship was identified between a large proportion of foreign capital and risk management time for *p* < 0.05 and rho = 0.34. This demonstrates the average power of the impact of experience in risk management on fears of too much foreign investments in the company’s operations. In turn, in terms of operational risk, it was stated that there was a significant relationship between risk management time and obsolete production facilities for *p* < 0.01 and rho = 0.36. This means that the wider the experience in risk management the more often the enterprises fear the technical condition of their production equipment. The identified significant statistical relationships allow for the conclusion that the enterprises handling risk management longer are able to better predict the upcoming threats and thus better prepare to deal with them.

To assess the relationship between the intensity of risk and the position responsible for risk management, the Pearson’s Chi-square test was applied. The summary results are presented in Table 7.

The results of testing the relationship between the assessment of intensity of risk and the position responsible for risk management in the company indicated significant relationships only in the area of market and economic risks. In the case of market risk, one may observe a significant relationship between the risk of loss of customers and the position for *p* < 0.0001 and χ^2^ = 0.206 and between stagnation in the market in the sector and the position for *p* < 0.0001 and χ^2^ = 0.450. A very low and low intensity of risk most often occurs where the company’s owner themselves or the people authorized deal with risk management, in turn, a very high and high frequency of this risk is observed when there is no person in the company responsible for the tasks related to the prediction and planning of risk prevention. This trend is observed both in the case of the risk of loss of customers and stagnation in the market. In the area of economic risk, significant relationships were observed between increase in taxes and compulsory contributions and the position occupied for *p* < 0.001 and χ^2^ = 0.403; poor availability of financial resources and the position occupied for *p* < 0.0001 and χ^2^ = 0.530 and increase in interest rates and the position occupied for *p* < 0.05 and χ^2^ = 0.328. A very low and low intensity of individual economic phenomena is most often observed when risk management is dealt with by a manager authorized by the management or a department head, in turn, a high level of risk is identified in the case when risk management is handled by the owner or a risk specialist.

## 5. Results

The summary of the obtained results of the extreme ratings broken down by the size of the company is presented in Table 8 and Table 9. The summary identifies the weakest and strongest risks of the occurrence of threats due to the pandemic from the point of view of the surveyed companies.

On the basis of the results contained in the tables, one may observe the identical key factors of market, economic and financial risk for the surveyed entities. All the surveyed enterprises, regardless of the number of employees and risk type, are most often threatened by strong competition in the sector, an increase in energy prices and insufficient profit of the company. At the same time, in the case of operational risk, for small and medium-sized enterprises, the greatest threat is incomplete use of production capacity, additionally for small ones, it is low innovation level. Regardless of the size of the surveyed entities, a high risk of unreliable suppliers, an increase in taxes and compulsory contributions and share of foreign capital in implemented investments due to the pandemic are rarely observed. The concerns of microenterprises are less likely to relate to unused production capacity in the period of economic slowdown. As a result of the conducted analysis of variance, it was indicated that the number of employees affects the level of intensity of market and operational risks, which enterprise of the SME sector are exposed to. At the same time, it was not demonstrated that the employment factor has an impact on financial and economic risks, which largely depend on the macroenvironment and, therefore, affect the examined sector of enterprises to a similar extent.

As a result of the conducted statistical testing, a few significant relationships between individual types of risk and the company’s experience in risk management and the position designated for this purpose were identified. On their basis, one may conclude that in terms of most factors posing a threat to the activities of the SME sector, experience in risk management is not relevant. This may be due to certain unpredictability and exceptionality of some phenomena like the COVID-19 pandemic, which requires the adjustment of the strategies and operations of enterprises to new circumstances. On the other hand, it was observed that this experience takes on particular importance in the case of the risk of loss of customers, increase in prices of energy, a large proportion of foreign capital, and obsolete machine park. At the same time, the position in the company is responsible for assessing the intensity of risk of loss of customers, stagnation in the market, increase in taxes and compulsory contributions, poor availability of financial resources, and increase in interest rates. Most often, high risk intensity was associated with a situation where there was no specific risk management position, in turn, the threats where risk management is handled by the company’s owner themselves or a specialist in this field were assessed more optimistically.

A major handicap in dealing with individual threats was a delayed response of enterprises to the economic slowdown which has occurred now as a result of the pandemic. Therefore, the gradual planning of activities and procedures, incorporated in business decisions of the SME sector, which, due to the scope of operations, rarely uses the advanced risk management systems, is crucial for handling various types of risks. The key issue in terms of risk management by enterprises of the SME sector is to develop appropriate business strategies, based on various scenarios of events in the business environment in relation to competitors in the market, the risk of an increase in prices of energy and raw materials and incomplete use of production capacity. Smaller enterprises, due to limited material resources, in order to limit the risk of strong competition, ought to use their intangible resources, which are unique and have a much greater impact on the strength of the competitive potential that is being built. Moreover, during the pandemic, social media as well as conducting business operations via the Internet are becoming an important tool in fighting competition. In order to improve the financial situation of enterprises during the pandemic, government support was also prepared in the form of subsidies for various purposes, which may also be used to minimize the costs associated with the acquisition of resources and energy. At the same time, the information on these programs is poorly disseminated so that only few enterprises benefit from them. It is one of the methods of dealing with the risk of rising prices of energy in the SME sector. At the same time, numerous available EU programs are designed to develop innovative activities, especially of the smallest enterprises, thus the risk of low innovativeness of small entities can be easily reduced by using government and EU subsidies for this purpose. It is also advisable to create and develop cooperation networks with the participation of SMEs, which effectively improves the level of involvement of enterprises of the sector in innovative activities and optimal use of production capacity, adjusted to the current market needs. In the case of the risk of insufficient profit, enterprises may also apply methods consisting in the use of financial ratio analysis, which demonstrates the deteriorating economic and financial situation of the company.

The ability to focus employees on the company’s objectives may also be helpful in the effective prevention or reduction of particular risks. In order to maintain the continuity of operations, it is also important to develop various scenarios in case one or more major suppliers fail to meet their obligations. During the pandemic, the company should also consider the relationships with employees and customers. The information on the basis of which business decisions are made should take into account both human and financial elements. An important part is also communication with the environment. All these activities are to create the early warning system, which requires considering the individual conditions of the operation of each enterprise.

## 6. Summary of Results

Due to the pandemic the SME sector is particularly threatened by an increase in the occurrence of business risk in the current conditions of government restrictions. In turn, the research conducted by P. Kokot-Stępień [77] confirms that the greatest threat to the activities of enterprises of the SME sector are external factors, that the surveyed entities have no actual impact on. The COVID-19 pandemic and its significant impact on activities of enterprises is the best example of the above [78]. This confirms the observations made in the article, according to which the risk of unpaid claims and inability to pay obligations among larger entities is increasing. In the abovementioned studies, the poor relationship of smaller entities with suppliers is also pinpointed, which becomes particularly important during the recession period.

At the same time smaller entities have a network of personal contacts with customers, which significantly affects the tightening and depriving business relationship of formalism and anonymity [79]. This, in turn, makes it easier for smaller operators to pick up market signal faster so that they more easily adapt to new conditions. At the same time, it is larger entities that better handle the management of economic risk, if only due to providing services to a wider market and greater opportunities of investments in new alternative solutions supporting the maintenance of the company. Moreover, appropriate risk management reduces various types of costs related to operation, material and supply, which makes it possible to gain a competitive advantage regardless of the size of the company and to survive the difficult period of stagnation [80].

In this research, it was indicated that the size of the entity does not affect the assessment of intensity of individual risks, whereas Junior A. [81] presents the differentiation of the impact of risk factors, depending on the size of the entity, which requires the use of various strategies for risk management. In larger entities, detailed management processes concerning risk are carried out, whereas, in smaller ones, risk management is divided into smaller projects in order to facilitate monitoring. The differences in the risk assessment of micro-, small and medium-sized enterprises suggest taking various strategies and tools to counteract the threat to business liquidity [82]. At the same time, this research indicated that, in selected cases of risks, the company’s experience in risk management takes on crucial importance. The appropriate approach resulting from both years of risk management and the appointment of specialized staff significantly reduces the impact of potential threats to the operations of small and medium-sized enterprises. For this purpose, it is also recommended to select and apply the optimal business model in which the key partnership with suppliers is the most important, ensuring joint counteracting the negative effects of the pandemic [83].

In addition, the conducted research allows for general conclusions concerning risk management in the SME sector during the pandemic. Enterprises ought to take actions to plan and prevent or eliminate the identified risks in the event of the economic slowdown. The risk management itself in Poland is insufficiently developed and disseminated, as a result of which many small enterprises do not have knowledge about available forms of support [84]. The exceptionality of the current economic situation causes that there are no ready and effective tools and methods to deal with the consequences of the pandemic, not to mention countermeasures prior to the emergence of related risks. Enterprises lack experience and practical guidance concerning the use of the risk management system in the current situation. Knowledge and experience in risk management builds the company’s resilience to risk that cannot be accurately predicted.

## 7. Conclusions

The capital base of micro-, small and medium-sized enterprises is not strong enough to avoid financial losses or bankruptcy in most of them during the difficult pandemic period. It is a particular group of enterprises, among which risk management is particularly important since smaller entities are more often threatened by the closure of activities. Effective predicting and coping with threats may minimize and even stop phenomena and events undesirable for business. At the same time, proper risk management requires from these entities the awareness, identification, planning, and counteracting threats, which they have not dealt with so far. Risk sources should be identified at each management level, properly taken into account, described and, above all, controlled. Polish enterprises lack practical guidelines concerning the use of the risk management system. The enterprises deal with risks in specific areas usually separately, irrespective of the overall systemic view of risk management in the company.

The analyzed sector of enterprises is characterized by a narrow scale of activities, thus, there is not always the need for risk management systems to be used by them. At the same time, these entities are able to assess the impact of individual phenomena on the company’s activities, however, a high rate of their impermanence suggests that they cannot use their knowledge in practice. The conducted research enabled the identification and assessment of the intensity of the impact of individual business risks on the course of business activities of micro-, small and medium-sized enterprises as a result of the outbreak of the COVID-19 pandemic. The key risk factors most threatening to the activities of most of the surveyed enterprises are strong competition in the sector, an increase in energy prices, insufficient profit of the company, and incomplete use of production capacity. These factors indicate the main direction of planning and organizing the risk management system in the SME sector. At the same time, many entities at risk of bankruptcy ought to consider the outbreak of the pandemic as a lesson for the future when planning and managing risks associated with the business activity in the conditions of high political and environmental volatility. At the same time, the research has some constraints. It was carried out among Polish enterprises and needs to be extended to other countries, which indicates the future direction of research.

## Figures and Tables

**Table 1:** Percentage of market risk intensity broken down by the size of the company.

Market Risk Factors	Risk Intensity					
	Low			High		
	Size of the Company					
	Micro-	Small	Medium	Micro-	Small	Medium
Loss of customers	21	19	20	41	51	52
Strong competition in the sector	18	19	12	51	54	53
Stagnation in the market	36	34	21	30	27	42
Unreliable suppliers	54	35	47	23	31	21

**Table 2:** Percentage of economic risk intensity broken down by the size of the company.

Economic Risk Factors	Risk Intensity					
	Low			Low		
	Size of the Company					
	Micro-	Small	Medium	Micro-	Small	Medium
Increase in taxes and compulsory contributions	48	45	40	16	19	23
Poor availability of financial resources (grants, loans)	34	36	36	28	26	21
Increase in interest rates	41	44	43	22	14	25
Increase in prices of all types of energy	26	24	19	40	41	33

**Table 3:** Percentage of financial risk intensity broken down by the size of the company.

Financial Risk Factors	Risk Intensity					
	Low			Low		
	Size of the Company					
	Micro-	Small	Medium	Micro-	Small	Medium
Insufficient profit of the company	25	13	19	63	64	66
Foreign capital (significant part of foreign capital)	40	32	52	23	25	27
Unpaid claims	23	25	27	51	46	45
Inability to pay obligations (insolvency)	22	24	28	54	52	39

**Table 4:** Percentage of operational risk intensity broken down by the size of the company.

Operational Risk Factors	Risk Intensity					
	Low			High		
	Size of the Company					
	Micro-	Small	Medium	Micro-	Small	Medium
Incomplete use of production capacity	54	17	25	15	57	45
Obsolete production facilities	52	59	41	16	11	11
Low level of innovation	47	42	55	19	21	23
Growing number of complaints	43	64	54	15	23	25

**Table 5:** The analysis of variance of intensity of individual business risks of the SME sector using the F test.

Type of Risk		Sum of Squares	df	MS	F	*p*-Value
**Market risk**	Between groups	0.45	2	0.0325	14.21	0.0063
	Within groups	1171.63	338	0.0403		
	Total	1177.27	342			
**Financial risk**	Between groups	1.60	2	0.0230	33.91	0.0571
	Within groups	631.15	263	0.0271		
	Total	632.62	275			
**Economic risk**	Between groups	0.005	2	0.0001	73.01	0.2383
	Within groups	5.70	272	0.0211		
	Total	6.49	271			
**Operational risk**	Between groups	2.06	2	0.0062	117.02	0.0310
	Within groups	464.13	163	0.0382		
	Total	475.41	171			

**Table 6:** The results of the research into the relationships between the intensity of risk and risk management time using the Spearman rank test.

Market Risk Factors	Loss of Customers	Strong Competition in the Sector	Stagnation in the Market	Unreliable Suppliers
Spearman’s rho coefficient value	0.45	0.21	0.22	−0.16
*p*-value	0.000	0.148	0.325	0.441
**Economic risk factors**	Increase in taxes and compulsory contributions	Poor availability of financial resources (grants, loans)	Increase in interest rates	Increase in prices of all types of energy
Spearman’s rho coefficient value	−0.17	0.23	0.15	0.32
*p*-value	0.309	0.100	0.478	0.008
**Financial risk factors**	Insufficient profit of the company	Foreign capital (significant part of foreign capital)	Unpaid claims	Inability to pay obligations (insolvency)
Spearman’s rho coefficient value	0.22	0.34	0.23	0.16
*p*-value	0.134	0.026	0.323	0.394
**Operational risk factors**	Incomplete use of production capacity	Obsolete production facilities	Low level of innovation	Growing number of complaints
Spearman’s rho coefficient value	0.13	0.36	0.21	0.20
*p*-value	0.651	0.003	0.171	0.187

**Table 7:** The results of the research into the relationships between the intensity of risk and the position responsible for risk management using the Pearson’s Chi-square test.

Market Risk Factors	Loss of Customers	Strong Competition in the Sector	Stagnation in the Market	Unreliable Suppliers
Pearson’s Chi-square value	0.206	0.285	0.450	0.321
*p*-value	0.000	0.097	0.000	0.055
**Economic risk factors**	Increase in taxes and compulsory contributions	Poor availability of financial resources (grants, loans)	Increase in interest rates	Increase in prices of all types of energy
Pearson’s Chi-square value	0.403	0.530	0.328	0.250
*p*-value	0.004	0.000	0.035	0.200
**Financial risk factors**	Insufficient profit of the company	Foreign capital (significant part of foreign capital)	Unpaid claims	Inability to pay obligations (insolvency)
Pearson’s Chi-square value	0.263	0.191	0.240	0.142
*p*-value	0.153	0.786	0.519	0.818
**Operational risk factors**	Incomplete use of production capacity	Obsolete production facilities	Low level of innovation	Growing number of complaints
Pearson’s Chi-square value	0.224	0.258	0.259	0.301
*p*-value	0.317	0.172	0.168	0.068

**Table 8:** Highest risk factors broken down by the size of the company and the percentage of extreme ratings during the COVID-19 pandemic.

Size of the Company	Market Risk	Economic Risk	Financial Risk	Operational Risk
Microentities	Strong competition in the sector	Increase in prices of energy	Insufficient profit of the company	Low level of innovation
Small entities	Strong competition in the sector	Increase in prices of energy	Insufficient profit of the company	Incomplete use of production capacity
Medium entities	Strong competition in the sector	Increase in prices of energy	Insufficient profit of the company	Incomplete use of production capacity

**Table 9:** Lowest risk factors broken down by the size of the company and percentage of extreme ratings during the COVID-19 pandemic.

Size of the Company	Market Risk	Economic Risk	Financial Risk	Operational Risk
Microentities	Unreliable suppliers	Increase in taxes and compulsory contributions	Foreign capital (significant part of foreign capital)	Incomplete use of production capacity
Small entities	Unreliable suppliers/stagnation in the market	Increase in taxes and compulsory contributions	Foreign capital (significant part of foreign capital)	Growing number of complaints
Medium entities	Unreliable suppliers	Increase in taxes and compulsory contributions	Foreign capital (significant part of foreign capital)	Growing number of complaints

## Data Availability

The data presented in this study are available on request from the corresponding author. The data are not publicly available due to privacy reasons.
